# Bilateral breast implant-associated anaplastic large cell lymphoma presenting as late peri-implant effusion: a case report

**DOI:** 10.1093/jscr/rjag917

**Published:** 2026-10-14

**Authors:** Noof Al Shaibani, Gala Kaazan Moussallem, Naina Simon, Omar R Gomaa, Khalid A Al Sindi

**Affiliations:** Surgical Oncology Department and Breast Unit, Bahrain Oncology Center, Road 2835, Block 228, Busaiteen 00228, Bahrain; Surgical Oncology Department and Breast Unit, Bahrain Oncology Center, Road 2835, Block 228, Busaiteen 00228, Bahrain; Histopathology Department, King Hamad University Hospital, Building 2435, Road 2835, Block 228, Busaiteen 00228, Bahrain; Surgical Oncology Department and Breast Unit, Bahrain Oncology Center, Road 2835, Block 228, Busaiteen 00228, Bahrain; Histopathology Department, King Hamad University Hospital, Building 2435, Road 2835, Block 228, Busaiteen 00228, Bahrain

**Keywords:** breast implant-associated anaplastic large cell lymphoma, BIA-ALCL, breast implants, peri-implant effusion, capsulectomy, PET-CT

## Abstract

Breast implant-associated anaplastic large cell lymphoma (BIA-ALCL) is a rare T-cell lymphoma strongly associated with textured breast implants; bilateral synchronous disease is exceptionally uncommon. We report a 51-year-old woman with 6 months of right breast swelling, 20 years after bilateral augmentation and 3 years after revision surgery. Imaging showed bilateral peri-implant effusions with irregular capsular thickening. Ultrasound-guided aspiration revealed malignant cells suspicious for high-grade lymphoma, and staging positron emission tomography-computed tomography (PET-CT) showed no distant disease. The patient underwent bilateral en-bloc capsulectomy with removal of both implants. Using the tumor, node, metastasis staging system for BIA-ALCL, the disease was classified as T3N0M0 (Stage IC) bilaterally. Histopathology confirmed bilateral BIA-ALCL with CD30-positive, anaplastic lymphoma kinase (ALK)-negative cells. Postoperative PET-CT showed patchy metabolic activity, but biopsy confirmed a foreign body giant cell reaction without residual malignancy. Bilateral BIA-ALCL should be considered with late peri-implant effusion even when symptoms are unilateral, and suspicious postoperative PET-CT findings require histological confirmation.

## Introduction

Breast implant-associated anaplastic large cell lymphoma (BIA-ALCL) is a rare non-Hodgkin T-cell lymphoma arising in the peri-implant capsule, strongly associated with textured breast implants [1]. It is characterized by large anaplastic cells with strong CD30 expression and anaplastic lymphoma kinase (ALK) negativity [1, 2]. Disease typically presents years after implantation as delayed peri-implant effusion, though capsular thickening, swelling, pain, asymmetry, a mass, or lymphadenopathy may also occur [2, 3].

Reported cases continue to increase worldwide, though true incidence remains difficult to define because of differences in implant exposure, reporting, and follow-up [4]. Most cases are unilateral; bilateral synchronous BIA-ALCL is exceptionally rare. Management relies largely on expert consensus, with complete surgical excision of implant and capsule considered the cornerstone of treatment for localized disease [3]. We report a rare case of bilateral BIA-ALCL in a 51-year-old woman presenting with unilateral swelling but bilateral peri-implant effusions.

## Case presentation

A 51-year-old woman presented with a 6-month history of progressive right breast swelling, 20 years after bilateral breast augmentation and 3 years after revision surgery for implant volume adjustment. Previous procedures were uneventful, and there was no relevant family history. On examination, the right breast was swollen with no palpable mass, nipple retraction, skin changes, or axillary lymphadenopathy.

Initial aspiration at an outside facility was interpreted as inflammatory exudate, and the patient was treated conservatively with antibiotics. Symptoms persisted, and she was referred for further evaluation. Breast magnetic resonance imaging (MRI) showed distension of the right peri-implant capsule by fluid compressing the implant, with irregular posterior capsular thickening bilaterally and no suspicious parenchymal mass or lymphadenopathy (Fig. 1). Ultrasound demonstrated a complex peri-implant fluid collection with internal debris and capsular irregularity, up to 1.7 cm in depth (Fig. 2). Ultrasound-guided aspiration yielded 20 ml of turbid fluid; cytology revealed malignant cells suspicious for high-grade lymphoma. Staging positron emission tomography-computed tomography (PET-CT) showed bilateral implants without FDG-avid nodal or distant disease (Fig. 3).

**Figure 1:**
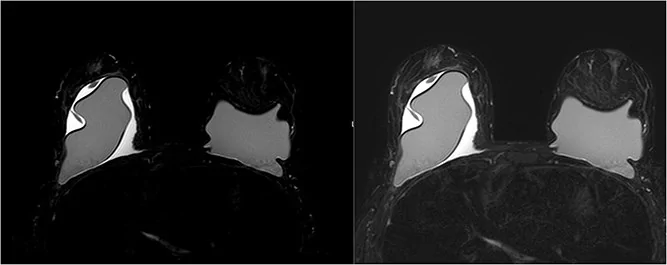
Breast MRI demonstrating bilateral peri-implant fluid collections, more prominent on the right side, with compression of the right breast implant and associated irregular capsular thickening.

**Figure 2:**
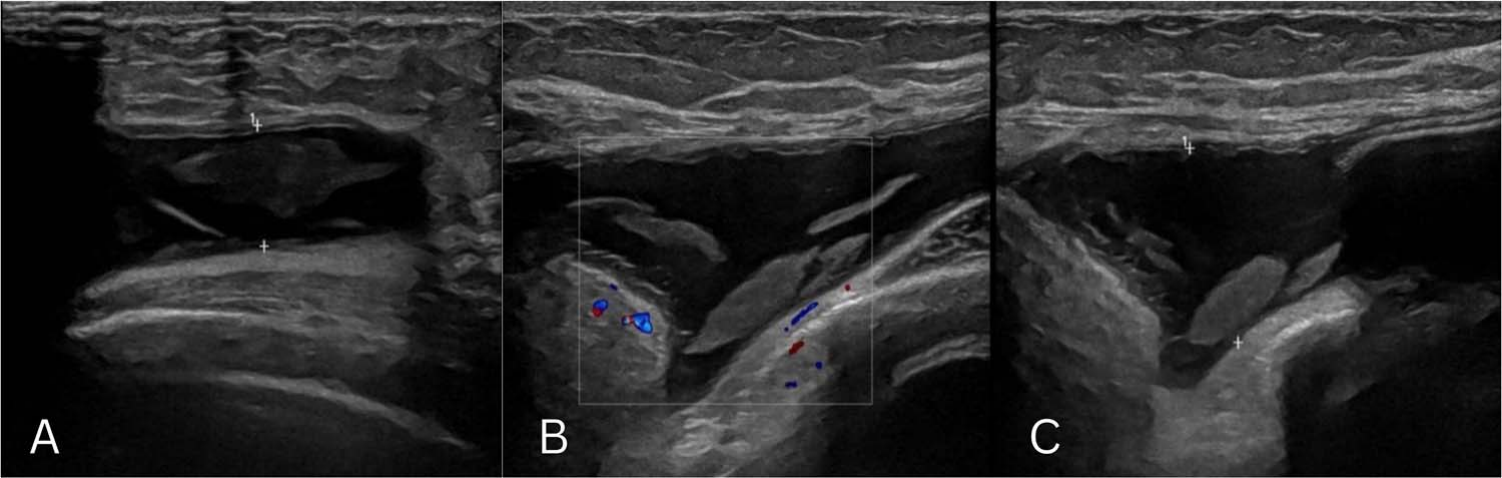
Breast ultrasound demonstrating a complex peri-implant fluid collection. (A) Grayscale ultrasound image showing peri-implant fluid with internal echogenic debris, measuring ~1.0 cm in depth. (B) Colour Doppler ultrasound image demonstrating the complex peri-implant collection without appreciable internal vascularity. (C) Additional grayscale image showing irregular peri-implant fluid collection with echogenic internal material, measuring ~1.68 cm in depth.

**Figure 3:**
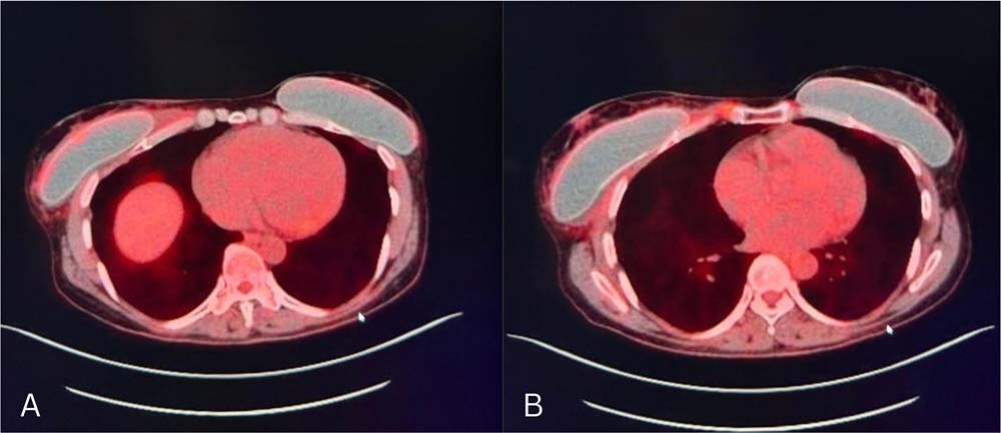
Preoperative staging PET-CT. (A and B) Selected axial fused PET-CT images demonstrating bilateral breast implants. The staging examination showed no evidence of fluorodeoxyglucose (FDG)-avid regional nodal or distant systemic disease.

The patient underwent bilateral en-bloc capsulectomy with removal of both implants. Intraoperatively, the right posterior capsule was nodular and adherent to the pectoralis muscle and chest wall; the left implant had a gelatinous appearance, with the left posterior capsule also adherent to the pectoralis muscle. Both capsules were excised as completely as possible (Fig. 4).

**Figure 4:**
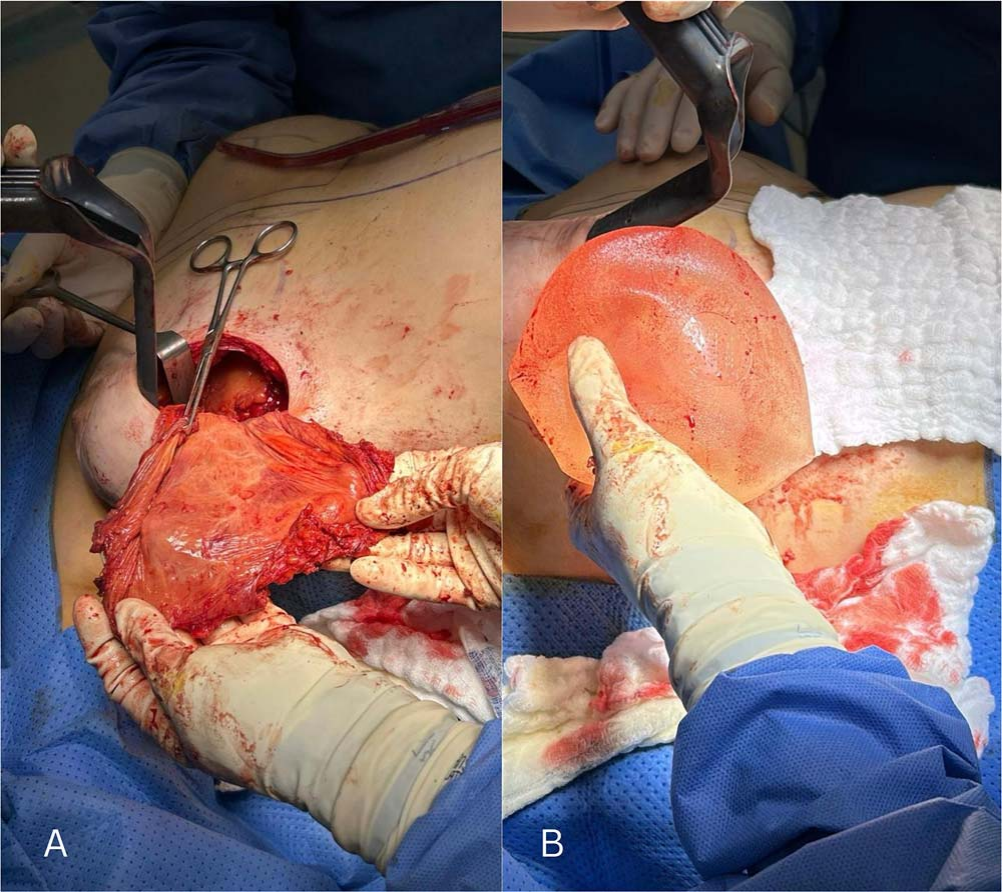
Intraoperative findings during capsulectomy. (A) Gross appearance of the posterior peri-implant fibrous capsule demonstrating diffuse nodularity and an irregular surface. (B) Intraoperative view of the breast implant *in situ*.

Microscopy showed infiltration by atypical large anaplastic cells with prominent nucleoli, including hallmark cells, arranged in small clusters and sheets within a fibrosclerotic stromal background, with no necrosis. One reactive lymph node was negative for malignancy (Fig. 5). Immunohistochemistry showed positivity for CD30, EMA, CD45, CD43, CD2, and CD4, and negativity for CD3, CD8, CD20, CD79a, PAX5, ALK-1, cytokeratin AE1/AE3, CD15, CD68, CD5, Ber-EP4, and CD34, confirming bilateral BIA-ALCL (Fig. 5).

**Figure 5:**
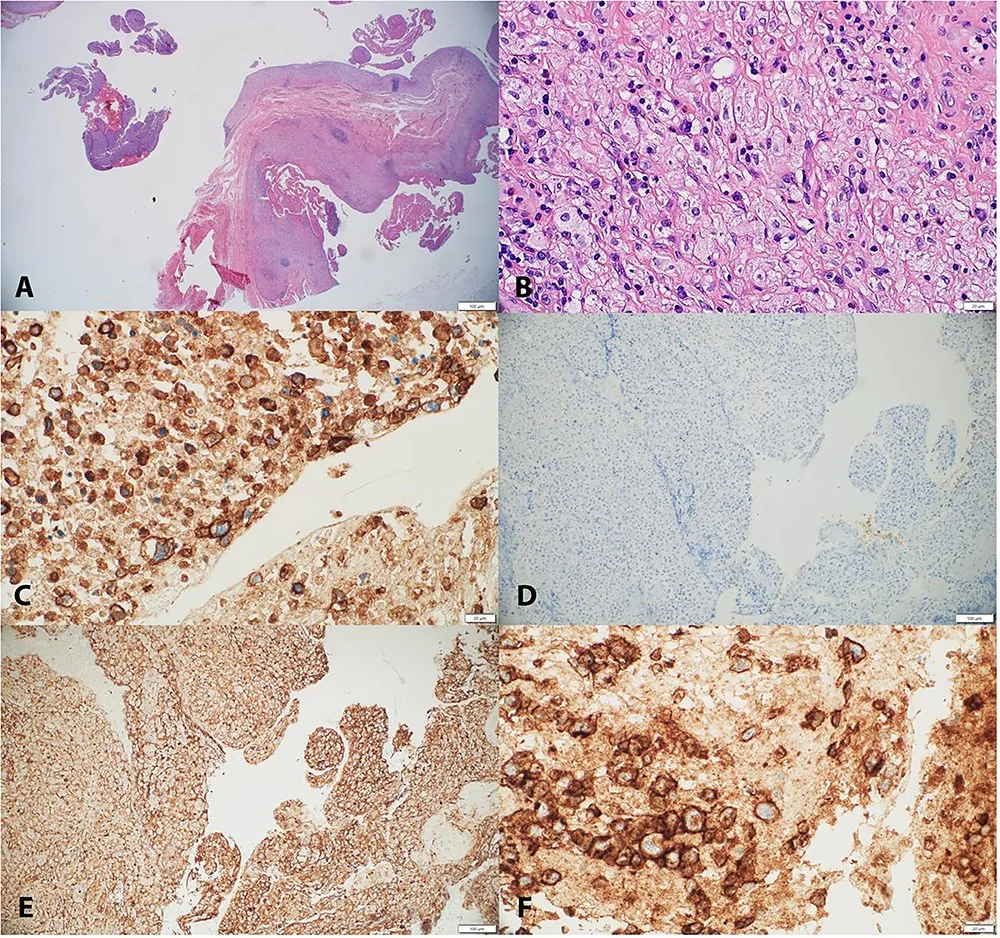
Microscopic examination of the peri-implant fibrous capsule stained with haematoxylin and eosin. (A) Low-power view showing infiltration of the fibrous capsule by atypical lymphoid cells. (B) Higher-power view demonstrating large atypical anaplastic cells within the fibrous capsule. Immunohistochemical staining of the peri-implant capsule. (C) The atypical large cells demonstrate strong positivity for CD30. (D) The atypical cells are negative for anaplastic lymphoma kinase (ALK), supporting the diagnosis of breast implant-associated anaplastic large cell lymphoma. Additional immunophenotyping of the peri-implant capsule. (E) The atypical cells demonstrate positivity for CD2. (F) The atypical cells demonstrate positivity for CD4.

Based on the tumor, node, metastasis staging system for BIA-ALCL, the disease was classified as T3N0M0 (Stage IC) bilaterally: infiltration was confined to the capsule by cell aggregates/sheets (T3), one lymph node was negative (N0), and staging PET-CT showed no distant disease (M0), despite the intraoperative impression of capsular adherence to muscle.

A follow-up PET-CT 1 month after surgery showed patchy metabolic activity in the residual right breast parenchyma, right pectoral and chest wall muscles, and left pectoralis fibres, raising concern for residual disease. Repeat biopsy showed a foreign body giant cell reaction with no malignancy, confirming this reflected postoperative inflammation rather than true extracapsular extension. The case was discussed in a multidisciplinary setting, and active surveillance was planned.

## Discussion

BIA-ALCL spans a spectrum from effusion-limited to infiltrative mass-forming disease, the latter carrying a worse prognosis [5]. Our patient presented with unilateral swelling, yet imaging revealed bilateral peri-implant effusions, underscoring that unilateral symptoms do not exclude bilateral disease.

Pathogenesis is multifactorial, involving textured implant surfaces, chronic inflammation, bacterial biofilm, and host immune factors [6]. Chronic antigenic stimulation likely drives lymphomagenesis and may explain why early presentations mimic benign inflammatory collections, as occurred here.

Ultrasound is the first-line imaging modality for detecting effusion and capsular abnormality and enabling image-guided aspiration [7]; MRI adds detail on capsular involvement, while PET-CT is used for staging [3, 7]. Pathological confirmation requires cytology, cell block preparation, and immunophenotyping, since cytomorphology alone is insufficient [8]; CD30 positivity must be interpreted in context, as it also occurs in reactive conditions [2, 8].

Bilateral synchronous BIA-ALCL is under-recognized when symptoms are unilateral, reinforcing the need for bilateral imaging, and histopathological examination of both capsules, since contralateral disease may be clinically silent.

Complete capsulectomy with implant removal is the mainstay of treatment for localized BIA-ALCL, with adjuvant therapy reserved for advanced or residual disease [3, 9, 10]. Consistent with American Association of Plastic Surgeons consensus data showing significantly better event-free and overall survival with complete excision of implant and capsule compared with partial removal or chemotherapy/radiation alone [3], both implants and capsules were excised here. Because the disease was staged T3N0M0 (Stage IC) rather than T4, with no histological evidence of extracapsular or nodal involvement, adjuvant systemic therapy was not indicated, and surveillance was adopted after biopsy excluded residual malignancy.

A key lesson is cautious interpretation of postoperative PET-CT, as postoperative inflammation and foreign body reaction can mimic residual disease. Here, suspicious uptake proved on biopsy to represent a foreign body giant cell reaction, supporting mandatory tissue confirmation before escalating to systemic therapy when postoperative imaging is equivocal.

## Conclusion

Bilateral BIA-ALCL is rare but should be considered in patients with breast implants presenting with delayed peri-implant effusion, even when symptoms are unilateral. This case highlights the importance of bilateral imaging and pathological assessment, complete surgical excision when feasible, and histological confirmation of suspicious postoperative PET-CT findings before initiating further treatment.
